# Isomer-Specific Branching Ratios in the Formation of Cyanopropene (C_3_H_5_CN) through the C_3_H_6_ + CN Reaction under Interstellar Conditions

**DOI:** 10.1021/acsearthspacechem.5c00274

**Published:** 2025-11-06

**Authors:** María Mallo, Marcelino Agúndez, Carlos Cabezas, José Cernicharo, Germán Molpeceres

**Affiliations:** 518690 Instituto de Física Fundamental, CSIC, C/Serrano 123, 28006 Madrid, Spain

**Keywords:** astrochemistry, gas-phase reactions, molecular clouds, chemical kinetics, reaction mechanisms

## Abstract

We investigated the reaction of propene (C_3_H_6_) with the cyano radical (CN) in light of the recent detection of five cyanopropene isomers in TMC-1. To provide reliable branching ratios, we characterized the stationary points on the potential energy surface using the high-accuracy jun-ChS-F12 method. The resulting energetics were then employed to derive temperature-dependent rate constants. Our calculations show that the formation of all observed cyano derivatives is feasible through this reaction, although it is secondary compared to the dominant formation channel of vinyl cyanide (C_2_H_3_CN). The predicted branching ratios are in good agreement with the observations, with the discrepancies prompting further investigation on the destruction mechanisms of the different isomers. Overall, this work supports a scenario in which these cyano derivatives in TMC-1 arise primarily from pure gas-phase chemistry.

## Graphical abstract

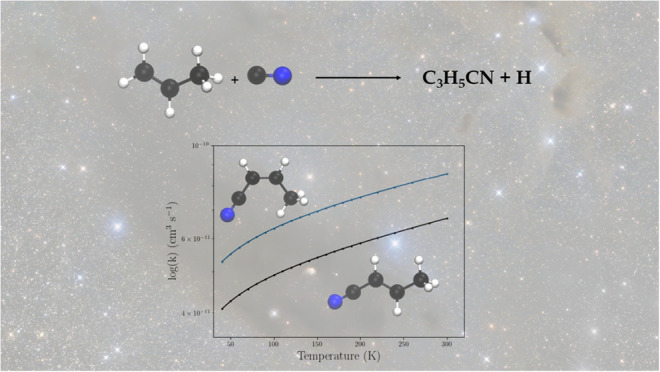

## 1 Introduction

A significant fraction of the molecules recently detected in the interstellar medium (ISM) contain a cyano group (R-CN) in their structure.^1–11^ This prevalence can be explained by two main factors. First, the CN radical is one of the most abundant species in the cold interstellar medium and exhibits high reactivity with unsaturated hydrocarbons, both aliphatic and aromatic. Second, the introduction of a cyano group strongly enhances the dipole moment compared with the parent hydrocarbon, which greatly facilitates the detection of these molecules through radioastronomical observations.

Propene (CH_3_CHCH_2_) is one of the most abundant hydrocarbons detected in cold dark clouds.^12^ The detection of propene came as a surprise because it is a partially saturated molecule, while most hydrocarbons detected in cold interstellar environments tend to be unsaturated. In any case, propene has a double C=C bond which makes it an ideal target to react with CN and C_2_H radicals since it is well established that CN and C_2_H react very efficiently with unsaturated hydrocarbons.^13^ Recently, five cyano derivatives of propene were detected in the cold dark cloud TMC-1 thanks to the QUIJOTE [Fn fn1] line survey,^14^ and the reaction between propene and CN arises as one of the msot likely formation pathways of these cyanated derivatives. The reaction between CN and C_3_H_6_ has been studied theoretically^15^ and experimentally, supporting the rapid reaction at low temperatures.^16–18^

One interesting aspect of the C_3_H_6_ + CN reaction is that it can lead to the formation of several isomers, both positional and stereoisomers. Every possible positional isomer and stereoisomer has been detected in TMC-1,^6^ including E-CH_3_CHCHCN, Z-CH_3_CHCHCN, CH_2_C­(CN)­CH_3_ + H, s-CH_2_CHCH_2_CN and g-CH_2_CHCH_2_CN + H. The reaction channels can therefore be defined as

$$\begin{array}{llll} C_{3}H_{6}+\mathrm{CN} & \to & E-{\mathrm{CH}}_{3}\mathrm{CHCHCN}+H & \left(1\right)\\ & \to & Z-{\mathrm{CH}}_{3}\mathrm{CHCHCN}+H & \left(2\right)\\ & \to & {\mathrm{CH}}_{2}C\left(\mathrm{CN}\right){\mathrm{CH}}_{3}+H & \left(3\right)\\ & \to & s-{\mathrm{CH}}_{2}{\mathrm{CHCH}}_{2}\mathrm{CN}+H & \left(4\right)\\ & \to & g-{\mathrm{CH}}_{2}{\mathrm{CHCH}}_{2}\mathrm{CN}+H & \left(5\right) \end{array}$$

To which the following competitive channels can be added:

$$\begin{array}{llll} C_{3}H_{6}+\mathrm{CN} & \to & {\mathrm{CH}}_{2}\mathrm{CHCN}+{\mathrm{CH}}_{3} & \left(6\right)\\ & \to & {\mathrm{CH}}_{2}{\mathrm{CHCH}}_{2}+\mathrm{HCN} & \left(7\right) \end{array}$$

The positional isomers that can be formed through the C_3_H_6_ + CN reaction also include the isocyano derivatives of propene, which have not been detected in the ISM, so far. However, they are considered in this work as they can be formed through the same reaction mechanism as

$$\begin{array}{llll} C_{3}H_{6}+\mathrm{NC} & \to & E-{\mathrm{CH}}_{3}\mathrm{CHCHNC}+H & \left(8\right)\\ & \to & Z-{\mathrm{CH}}_{3}\mathrm{CHCHNC}+H & \left(9\right)\\ & \to & {\mathrm{CH}}_{2}C\left(\mathrm{NC}\right){\mathrm{CH}}_{3}+H & \left(10\right)\\ & \to & s-{\mathrm{CH}}_{2}{\mathrm{CHCH}}_{2}\mathrm{NC}+H & \left(11\right)\\ & \to & g-{\mathrm{CH}}_{2}{\mathrm{CHCH}}_{2}\mathrm{NC}+H & \left(12\right)\\ C_{3}H_{6}+\mathrm{CN} & \to & {\mathrm{CH}}_{2}\mathrm{CHNC}+{\mathrm{CH}}_{3} & \left(13\right)\\ & \to & {\mathrm{CH}}_{2}\mathrm{CHCH}+\mathrm{HNC} & \left(14\right) \end{array}$$

Assessing the relative importance the channels 1–7 (referred to in the text as RXN1-RXN7) is crucial to determine whether the presence of cyano-propene isomers in TMC-1 can be solely attributed to their formation through the title reaction, or whether additional mechanisms are required to reproduce the observed abundances. Moreover, the detection of different isomers with large energy differences provides key information on the role of kinetics against thermodynamics. The large number of possible products for the title reaction, including several stereoisomers, makes an experimental determination of branching ratios and rate constants particularly challenging. In this context, computational chemistry, combined with kinetic analyses based on statistical theories, offers a valuable approach. However, the C_3_H_6_ + CN system is medium-sized, and the application of the most accurate quantum chemical methods is computationally demanding. This contrasts with the need to achieve subchemical accuracy (i.e., better than 1 kcal mol^–1^) in order to resolve isomer-specific reaction channels. Recently, new composite methods have reached this level of accuracy for systems of comparable size.^19,20^ In this work, we present a detailed kinetic analysis of the C_3_H_6_ + CN reaction using these advanced composite approaches. Our astrochemical goal is to establish whether gas-phase chemistry via the title reaction can account for the observed abundances of cyano-propene isomers in TMC-1, or whether additional chemical pathways must be invoked. This question is particularly relevant in light of observations showing that the different isomers are present with abundances differing only by factors of 2–3,^6^ while at the same time, the energy separation between the isomers are high enough to expect much larger differences in the Boltzmann ratios at the low temperatures of TMC-1 (10 K).

The paper is organized as follows. In Section 2, we describe the computational methods employed to explore the potential energy surface (PES) of the C_3_H_6_ + CN reaction, as well as the kinetic framework used to derive rate constants and branching ratios. Section 3 presents the results of our quantum chemical calculations, including the characterization of stationary points on the PES and the outcomes of the kinetic simulations. In Section 4, we compare our findings with previous theoretical studies and with recent Quijote observations, highlighting possible avenues to improve the predictive power of our work and the follow-up calculations required to refine the branching ratios derived here. Finally, a brief summary is provided in Section 5.

## 2 Methodology

The goal of this article is to obtain rate constants and, more specifically, branching ratios accurate enough to allow us to discern isomer-specific reaction channels for the title reaction. Because conformers and stereoisomers of interstellar molecules can differ by merely a fraction of a kcal mol^–1^, as for example, for cyanomethanimine,^21^ a method capable of achieving subchemical accuracy is required for our purposes. The title reaction involves a medium-sized system (with a maximum of 11 atoms in the potential energy surface wells), and therefore, the most accurate composite methods, like the HEAT protocol^22^ are beyond our computational capabilities. In recent years, cheaper alternative highly accurate methods have been developed for the computation of accurate rate constants in the gas phase.^19^ In this work, we use the jun-ChS-F12 composite scheme.^20^

15 $$U=E_{\mathrm{el}}+E_{a‐\mathrm{ZPVE}}$$

where *E*_el_ is the electronic energy and *E*_a‑ZPVE_ is the anharmonic zero-point vibrational energy correction. *E*_el_ is calculated on top of the optimized geometries at the revDSD-PBEP86­(D3BJ)/jun-cc-pVTZ level^23–26^ as[Fn fn2]

16 $$E_{\mathrm{el}}=E_{\left(U\right)\mathrm{CCSD}\left(T\right)‐F12b/j‐\mathrm{TZ}}+\Delta E_{\mathrm{MP}2‐F12}^{\mathrm{CBS}}+\Delta E_{\mathrm{MP}2‐F12}^{\mathrm{CV}}$$

In the above equation, *E*_(U)CCSD(T)‑F12b/j‑TZ_ is the spin-unrestricted coupled-cluster energy with the inclusion of explicitly correlated F12 terms.^27^ Then, Δ*E*_MP2‑F12_^CBS^ + Δ*E*_MP2‑F12_^CV^ represent the complete basis-set and core–valence correction terms, calculated as described in the original reference. All correlated calculations use a restricted (open) Hartree–Fock wave function as a reference. The *E*_a‑ZPVE_ term is calculated using the fundamental frequencies from an unsupervised Vibrational Perturbation Theory to second order (VPT2) approach.^28,29^ The determination of *E*_a‑ZPVE_ is done at the revDSD-PBEP86­(D3BJ)/cc-pVTZ level, preoptimizing the structures using the smaller basis set before the vibrational analysis.

The codes used for the electronic structure calculations are as follows. We employed Gaussian16 for geometry optimization, PES scans, and vibrational calculations.^30^ The energy calculations required for the evaluation of the *E*_(U)CCSD(T)‑F12b/j‑TZ_, Δ*E*_MP2‑F12_^CBS^ and Δ*E*_MP2‑F12_^CV^ are performed using Molpro2022.2.^31,32^

### 2.1 Kinetic Analysis

The branching ratios for the title reaction can be determined as

17 $${\mathrm{BR}}_{i}=\frac{k_{i}}{\sum_{j}k_{j}}$$

where *k* represents the rate constant for a particular reaction channel (RXN1-RXN7). Phenomenological rate constants for each reaction channel were obtained using ab initio transition state based master equation (AITSME) under a microcanonical formalism. Each unimolecular reaction step is modeled using the Rice-Ramsperger-Kassel-Marcus (RRKM) theory where the sum and density of states is obtained based on the revDSD-PBEP86 rotational constants and anharmonic vibrational frequencies. Quantum tunneling is accounted for, using an Eckart fit of the ZPVE corrected barrier. Barrierless association reactions and back-dissociations are modeled using classical capture theory with a potential form *V*(*r*) of the type:

18 $$V\left(r\right)=-\frac{C_{6}}{r^{n}}$$

with *C*_6_ and *n* fitting constants of the long-range attractive potential. The *V*(*r*) points are obtained from relaxed potential energy scans at the revDSD-PBEP86­(D3BJ)/jun-cc-pVTZ. The final fit to derive *C*_6_ and *n* is done between 4–25 Å. Finally, symmetry numbers (σ) are included in our simulations. The individual rate constant for each process, barrierless or not, are then used to derive global rate constants using chemical simulations and a master equation formalism.^33,34^ All master equation calculations were performed employing the MESS code.^34^ The rate constants for each bimolecular channel are finally used to determine each individual BR_i_ and fitted to the usual Arrhenius-Kooij equation:

19 $$k=\alpha {\left(\frac{T}{300\;K}\right)}^{\beta }\;\exp\left(-\frac{\gamma }{T}\right)$$

that we later introduce in our own astrochemical rate equation models (Supporting Information).

## 3 Results

In the following, the reaction mechanism of C_3_H_6_ + CN is shown in detail in Sections 3.1 (association reaction), 3.2 (isomerizations) and 3.3 (abstraction reactions). Subsequently, the results of the kinetic research are reported in Section 3.4.

### 3.1 Association between CN and C_3_H_6_

The key requirement for the viability of the title reaction at low temperatures is the presence of barrierless entrance channels. At 10 K, this necessitates a barrierless formation of the CN-C_3_H_6_ complex. To verify whether this is the case in this reaction, we explored the long-range interaction potential for CN attacking the unsaturated carbon atoms of C_3_H_6_, specifically the −CH_2_ (**R1**) and –C­(H)– (**R2**) moieties. Figure 1 presents one-dimensional potential energy scans at the revDSD-PBEP86­(D3BJ)/jun-cc-pVTZ level for the capture of the CN radical. Moreover, and as evinced by a subsequent investigation of H-abstraction channels, we also perform a scan for RXN7, which is detailed in Section 3.3. A visual inspection of the potential energy for all channels confirms a barrierless profile, ensuring the feasibility of the C_3_H_6_ + CN reaction at low temperatures. The fit of the energetic profile is also shown in Figure 1, with numerical values of the fit shown in Table 1. A good fit was obtained assuming a r^6^ trend, which is characteristic of systems interacting at long-range through dispersion interactions.

**Figure 1:**
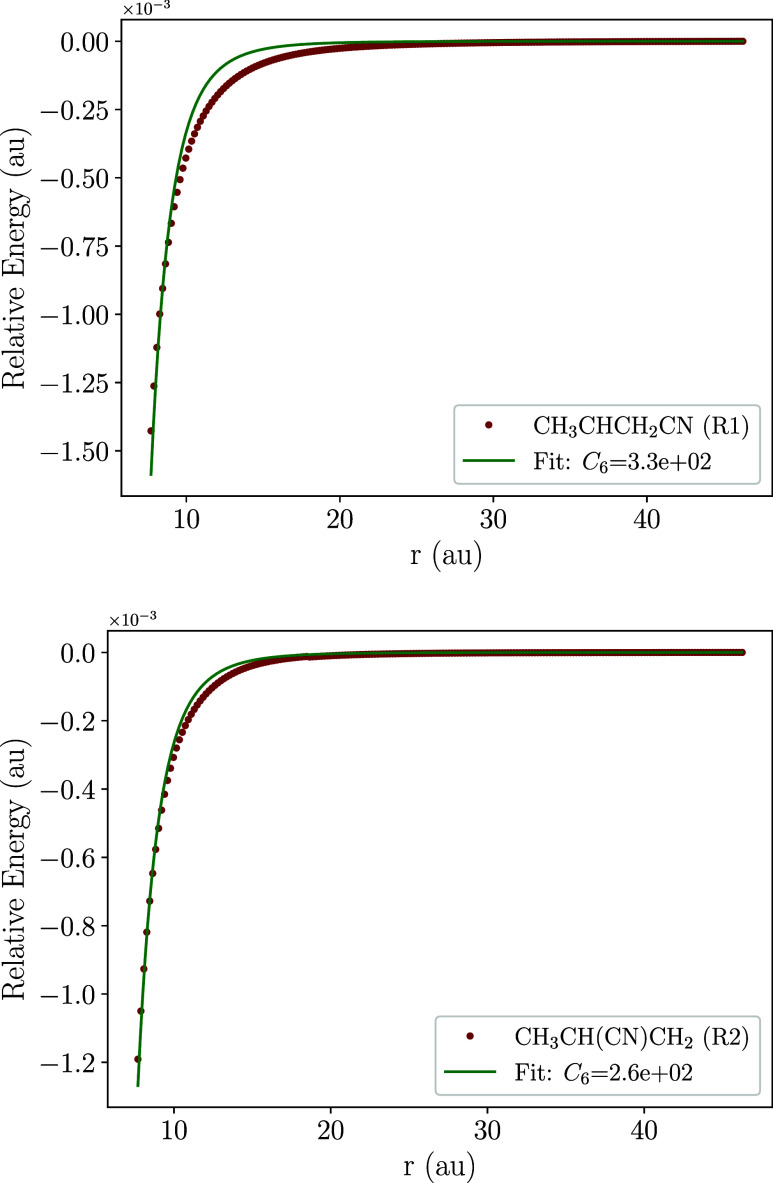
Energy profile for the long-range capture of C_3_H_6_ + CN.

**Table 1:** Values of Factor *n* and *C*_6_ for the Barrierless Association Reactions

radical	*n*	*C*_6_ (au)
CH_3_CHCH_2_CN	6	330.39
CH_3_CHCNCH_2_	6	264.35

We also consider the formation of isocyano adducts, CH_3_CHCH_2_NC (**R3**) and CH_3_CH­(NC)­CH_2_ (**R4**), in our calculations. The potential energy scans reveal the presence of short-range kinetic barriers that preclude the viability of the reaction at low temperatures. We note that the computed barriers for the formation of **R3** and **R4** are relatively small, at 1.6 and 0.9 kcal mol^–1^, respectively. In particular, the latter falls on the upper end of the uncertainty of our electronic structure method. While this might suggest the possibility of an isocyano product, specifically at the CH_2_ moiety (**R4**), further evolution of **R4** predominantly favors isomerization over H– or −CH_3_ elimination, as all elimination pathways exhibit energy barriers (see Section 3.2).

In a previous study on this reaction by Huang et al.^15^ the authors reported additional collision complexes, in this work referred to as association complexes. These include 3-member and 4-member ringed nitriles and isonitriles (**R5–R8**). In this work, we could not find them to proceed directly without barriers with the scanned reaction coordinates favoring always **R1** and **R2**. Therefore, they are formed by the isomerization of the more stable complexes (**R1–R4**). Furthermore, we report the presence of five isomeric forms (**R9–R13**) that result from the migration of an hydrogen atom within the adducts **R1** and **R3** and another two stationary points related with the barrierless hydrogen-abstraction (**R14–R15**), which are explained later in the text (Section 3.3).

### 3.2 Reactive Potential Energy Surface. Stationary Points

Due to the complexity of the reaction profile, the complete view of the stationary points is separated in three different representations: one for the cyano-addition isomers, another for the isocyano isomers and the last one for a barrierless hydrogen-abstraction process. The first two are shown in Figure 2 and the last one is detailed in Section 3.3.

**Figure 2:**
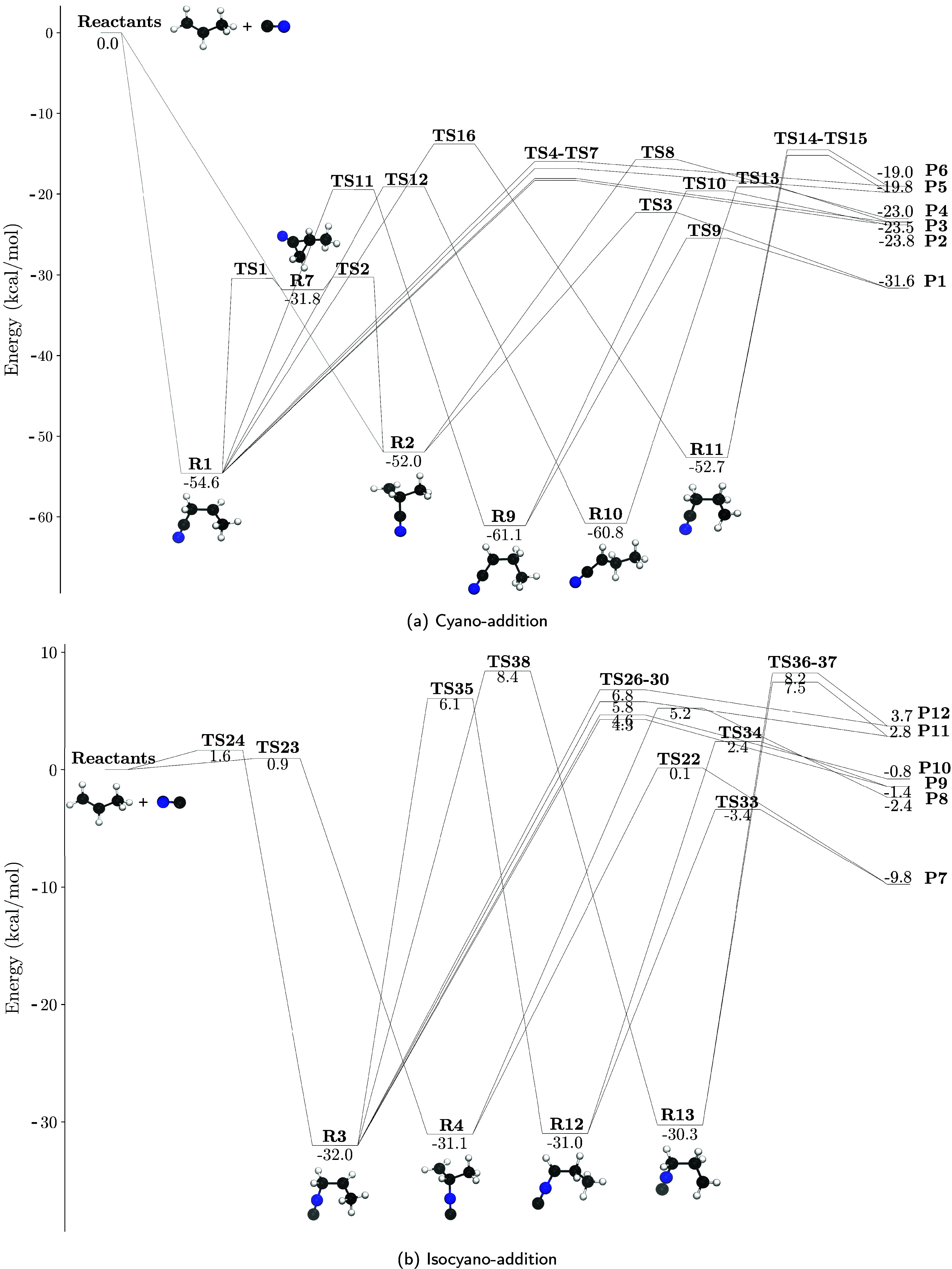
Reaction pathways of C_3_H_6_ + CN. The jun-ChS-F12 relative energies with the anharmonic revDSD/cc-pVTZ ZPE corrections are given in kcal mol^–1^. The potential wells **R5**, **R6** and **R8** and transition states **TS17-TS21** are not included in the energetic profiles because they interconnect cyano and isocyano isomers but all of them are listed in Table S1 (Supporting Info). The energetic values of all stationary points are listed in Table S1 (Supporting Info).

It is reasonable to consider whether it makes sense to decouple the cyano and isocyano reaction profiles. This approximation can only be done if the energetics reveal that the isocyano wells and products will be marginal in comparison with the cyano ones. Looking at the competition between cyano and isocyano isomers (Figure 2a,b) following capture in the cyano wells **R1** and **R2**, our findings indicate that the formation of isocyano compounds is unlikely from an energetic standpoint. Three key factors support this conclusion. First, all isocyano entrance channels exhibit barriers, as shown in Section 3.1. While these barriers are relatively small, they hinder direct association into **R3** and **R4**, particularly at the low temperatures of TMC-1 (10 K). Second, all exit barriers to isocyano channels are also emerged, and in most cases significantly so, except for the formation of C_2_H_3_NC, which has a low barrier (0.1 kcal mol^–1^). In third place, the barrier for isocyano to cyano isomerization is submerged (TS21 at −0.8 kcal/mol, included in Table S1 of the Supporting Information), therefore prevailing the isomerization to the cyano form over the evolution to isocyano products.

**Figure 3:**
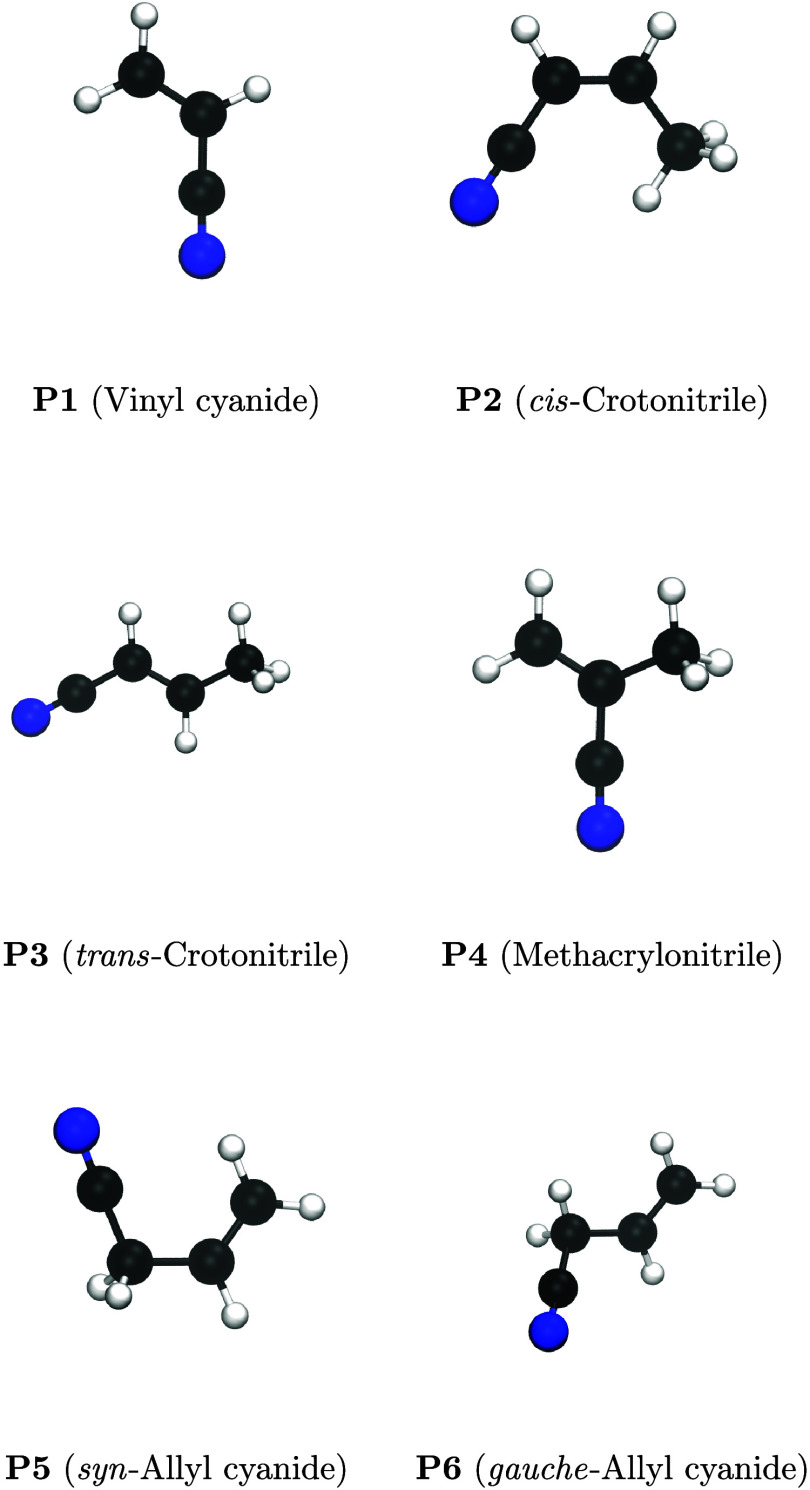
Schematic representation of the cyano-addition products.

However, considering the third factor, the high barriers for isocyano to cyano isomerization, it is reasonable to assume that the reaction will predominantly yield cyano products (**P1–P6**, Figure 3). Taking all these factors into account, we can confidently conclude that the contribution of isocyano products (**P7–P12**, Figure 4)­will be negligible in this reaction, and they are therefore excluded from any further analysis.

**Figure 4:**
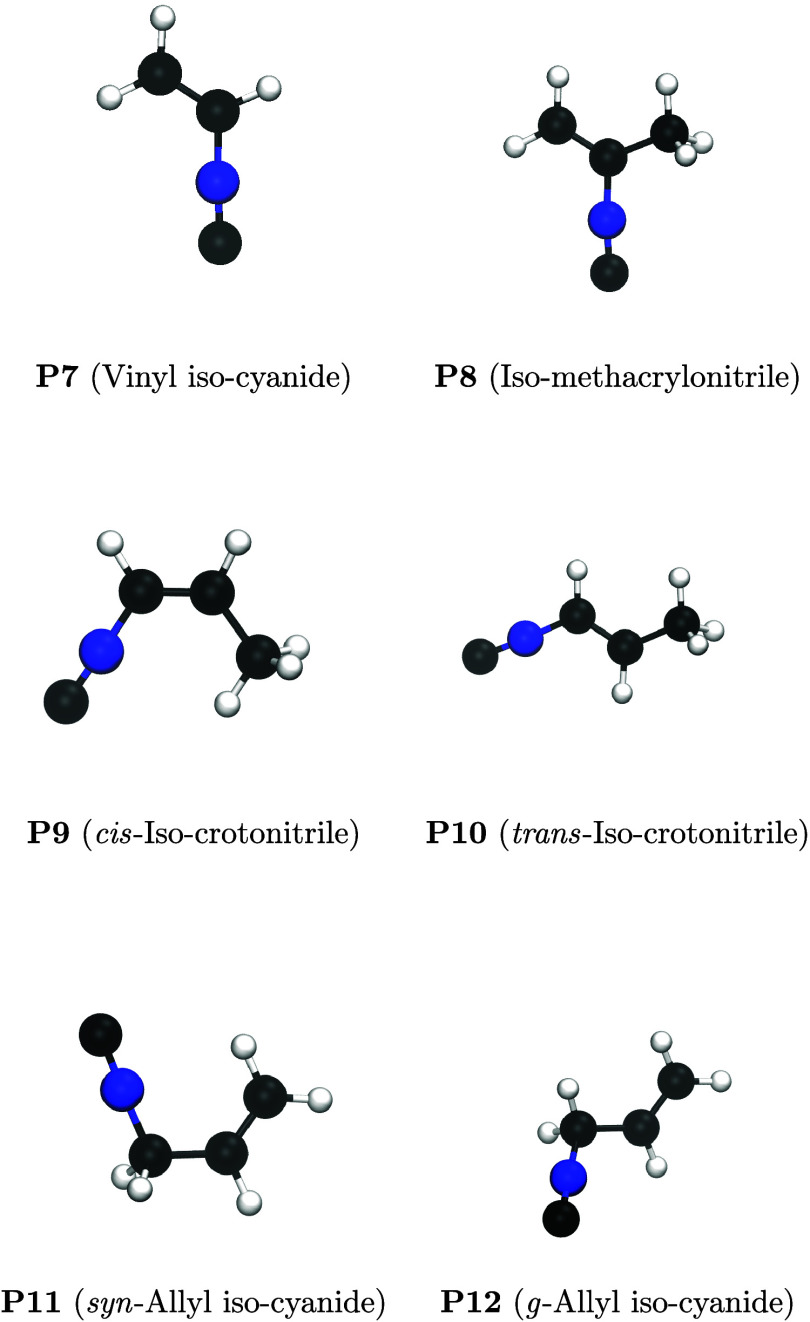
Schematic representation of the isocyano-addition products.

We coarse-grain the total PES based only on the stationary points that are relevant for the kinetic analysis, remaining only the reaction profile portrayed in Figure 2a and additional barrierless H-abstraction channel (Section 3.3). The association complexes, **R1** and **R2** serve as local minima of our PES, with energies around −50 kcal mol^–1^, indicating that this excess energy can be used to overcome other submerged barriers leading to the main cyano reaction products (**P1–P6**). The evolution from **R1** proceeds either via direct H elimination to products **P2**, **P3**, **P5**, and **P6** (isomers of the molecule generically referred to as cyanopropene, C_3_H_5_CN), or through CH_3_ elimination to form **P1** (vinyl cyanide, CH_2_CHCN). Similarly, the evolution from the **R2** intermediate follows an equivalent pathway; however, in this case, H elimination leads only to product **P4**, as other possible products are not energetically competitive. The elimination of CH_3_, however, remains viable (and dominant, Section 3.4) in **R2**. We also discard any exit channel arising from the cyclic intermediates.

After capturing CN in either **R1** or **R2**, the system can undergo isomerizations between them through the triangular intermediate **R7**. However, such an isomerization slightly favors **R1** owing to its higher stability. Apart from the back and forth isomerization, either **R1** and **R2** can evolve through fragment elimination, H or CH_3_ passing through submerged transition states, as mentioned above, (**TS3**–**TS7**) in a relatively narrow energy band (from −22.3 to −16.0 kcal mol^–1^).

If the system captures in **R1**, it can also suffer from tautomerization, which consists on relocating an hydrogen atom to form either **R9** (*cis*-CH_3_CH_2_CHCN) and **R10** (*trans*-CH_3_CH_2_CHCN) or **R11** (CH_2_CH_2_CH_2_CN) going through submerged transition states (**TS11**, **TS12** and **TS16**). These intermediates can then evolve to the main products, except for methacrylonitrile (**P4**), which can only be formed from **R2**.

The formation of products through such energetically close transition states has an important impact in the kinetics (Section 3.4) and highlights the importance of subtleties in the kinetic evolution of the system, such as the density of states at the transition states or the competition between H atom tunneling and CH_3_ elimination. Thanks to the energetic distribution of the transition states, we find that all the cyano products (**P1**-**P6**) are viable. This includes **P4**, whose presence in previous studies could not be confirmed^15^ and aligns our theoretical results with the expected behavior arising from observational evidence.^6^

Among the cyano-addition products, five isomeric forms of cyanopropene are present. The formation of *cis* (**P2**) and *trans*-crotonitrile (**P3**) arises from the elimination of different hydrogen atoms in the CH_2_ moiety, while *syn* (**P5**) and *gauche*-allyl cyanide (**P6**) result from H-elimination migration at the CH_3_ group. The exit barriers for *cis*/*trans*-crotonitrile differ by merely 0.2–0.5 kcal mol^–1^, whereas for *syn*/*gauche*-allyl cyanide, the difference is 0.7–0.9 kcal mol^–1^. These small energy differences, especially considering the energetic budget from the most stable well (−61.1 kcal mol^–1^, corresponding to **R9**), translate into similar branching ratios for the product isomers with further details in Section 3.4. A similar trend can be observed for iso-cyano products; however, as previously mentioned, they are excluded from subsequent kinetic analysis.

### 3.3 H-Abstraction Reactions

We also considered the possibility to form hydrogen cyanide (HCN) and hydrogen isocyanide (HNC) as an alternative to the CN and NC addition reactions. We sampled the following reactions:

$$\begin{array}{llll} C_{3}H_{6}+\mathrm{CN} & \to & {\mathrm{CHCHCH}}_{3}+\mathrm{HCN}/\mathrm{HNC} & \left(20\right)\\ & \to & {\mathrm{CH}}_{2}{\mathrm{CCH}}_{3}+\mathrm{HCN}/\mathrm{HNC} & \left(21\right)\\ & \to & {\mathrm{CH}}_{2}{\mathrm{CHCH}}_{2}+\mathrm{HCN}/\mathrm{HNC} & \left(22\right) \end{array}$$

The energetic descriptors for the abstraction reactions are gathered in Table 2. We found all reactions to be exothermic, with a clear gap in exothermicities between HCN and HNC formation. With respect to the activation energies (Δ*U* ^‡^), the vast majority of reactions present barriers that preclude their occurrence at low temperatures. We only find an open entrance channel for the formation of CH_2_CHCH_2_ + HCN confirmed through downhill intrinsic reaction coordinate (dIRC) calculations hinting at the absence of an energy barrier to abstract an hydrogen atom, so this reaction becomes a competitive channel to the cyano-additions in Figure 2a and it is considered in the subsequent kinetic analysis. The absence of an activation barrier for the formation of CH_2_CHCH_2_ can be attributed to the high thermodynamic stability of the resulting allyl radical, which is strongly stabilized by resonance.

**Table 2:** Activation (Δ*U* ^‡^) and Reaction (Δ*U* ^*R*^) Energies (in kcal mol^–1^) for H-Abstraction Processes

product	Δ*U* ^‡^	Δ*U* ^*R*^
*cis*-CH_3_CHCH + HCN	1.2	–15.3
*trans*-CH_3_CHCH + HCN	1.0	–15.8
CH_3_CCH_2_ + HCN	2.5	–19.5
CH_2_CHCH_2_ + HCN	–0.3	–39.7
*cis*-CH_3_CHCH + HNC	9.1	–0.7
*trans*-CH_3_CHCH + HNC	8.3	–1.1
CH_3_CCH_2_ + HNC	6.3	–4.9
CH_2_CHCH_2_ + HNC	6.0	–25.0

The reaction pathway corresponding to RXN 7 is shown in Figure 5, where we can see that the system evolves directly from the submerged prereactant complex (**R14**) to the abstraction products **P13** (HCN + CH_2_CHCH_2_). The barrierless nature of the capture process leading to **R14** is further illustrated in Figure 6. After the abstraction, we find a HCN–CH_3_CHCH_2_ complex after which we consider either an HCN addition to the CH_2_ moiety in a sort of roaming mechanism or evolve to the pure abstraction product barrierlesly (HCN + CH_3_CHCH_2_). In our case, the prereactant complex (**R14**) can experience roaming, forming the intermediate **R15** and eventually leading to the *gauche* isomer of cyanopropene (**P6**) through submerged transition states.

**Figure 5:**
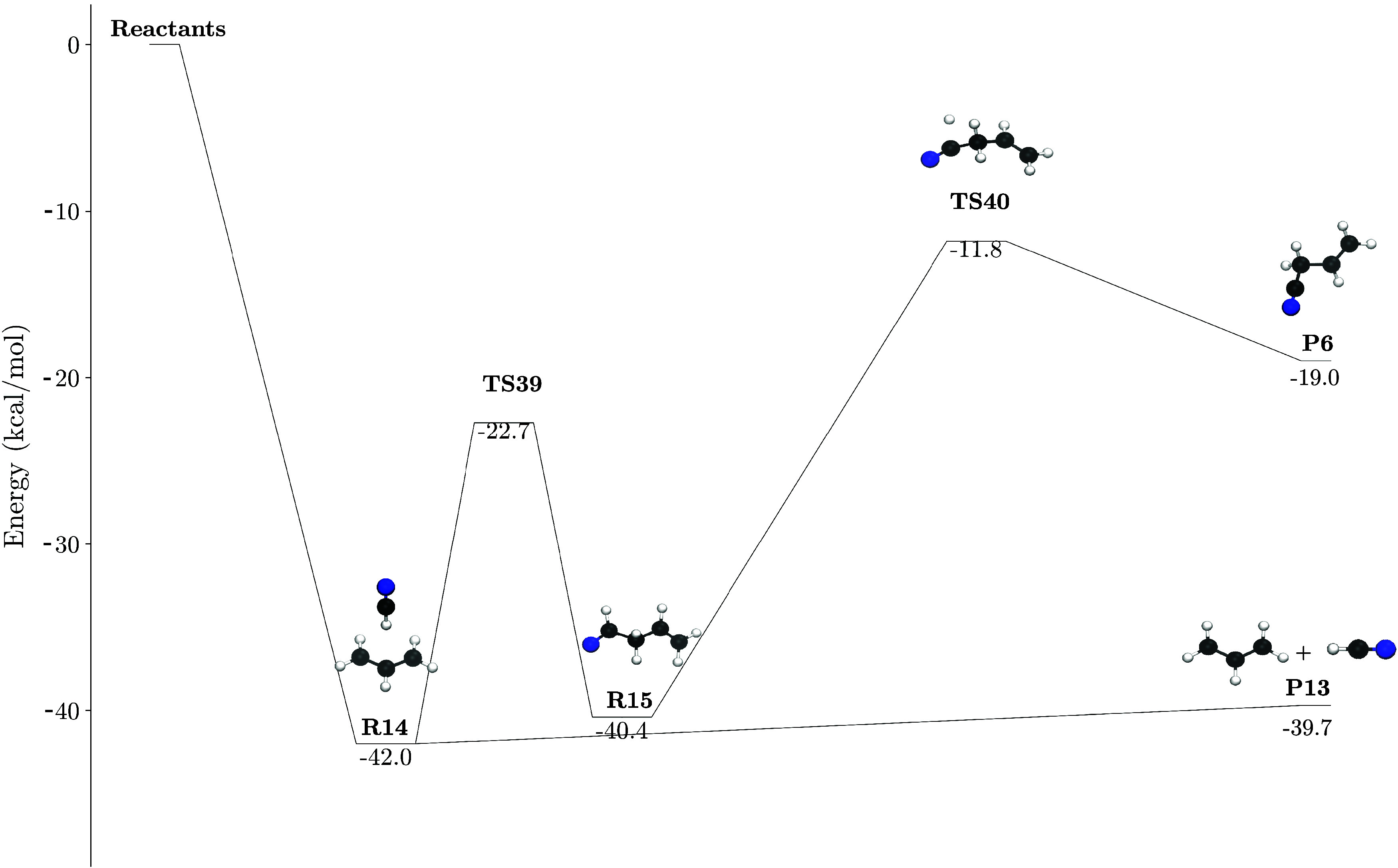
Energy profile for the H-abstraction reaction. The relative energies are given in kcal mol^–1^ and the zero-point vibrational energy (ZPVE) corrections are included.

**Figure 6:**
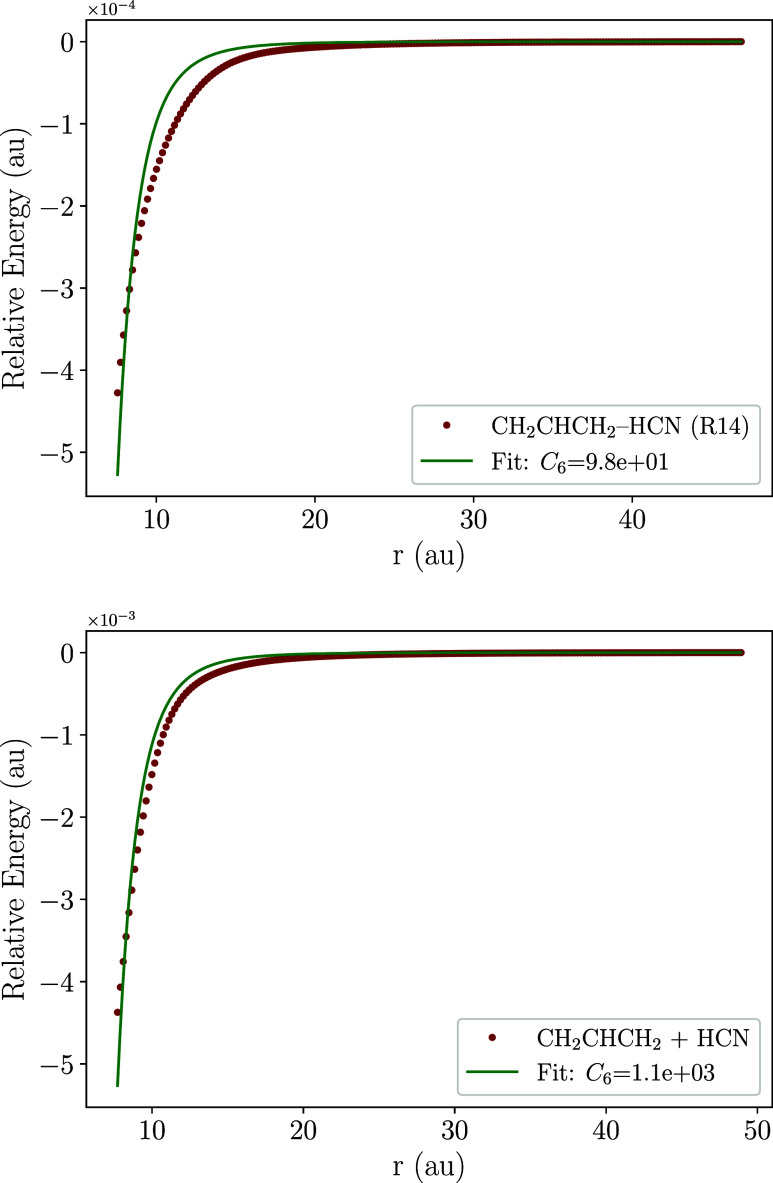
Energy profile for the long-range capture of (top panel) C_3_H_6_ + CN leading to H-abstraction and (bottom panel) CH_2_CHCH_2_ + HCN from **P13**. The capture coefficient (*C*_6_) from reactants to **R14** is 98.2 a.u whereas the capture of HCN in **P13** is 1116.4 a.u.

### 3.4 Kinetic Analysis

The phenomenological rate constants for the formation of the cyano-addition products (**P1–P6**) and the abstraction product (**P13**) were computed using the methodology described in Section 2.1 for a residual pressure of 10^–7^ atm and a temperature range of 40–300 K. The results are summarized in Table 3 and represented in Figure 7, where it is revealed that the formation of vinyl cyanide (CH_2_CHCN) with elimination of CH_3_ (**P1**) is dominant, with a rate constant of 8.87 × 10^–10^ cm^3^ s^–1^ at 40K and a branching ratio of 66.1%. The formation of CH_2_CHCN as major product of the reaction C_3_H_6_ + CN is consistent with the observational fact that vinyl cyanide is more abundant than the five cyanopropene isomers in TMC-1,^6^ although it is worthwhile to mention that vinyl cyanide can be formed from other reactions, like, for example C_2_H_4_ + CN → CH_2_CHCN + H.^16^

**Figure 7:**
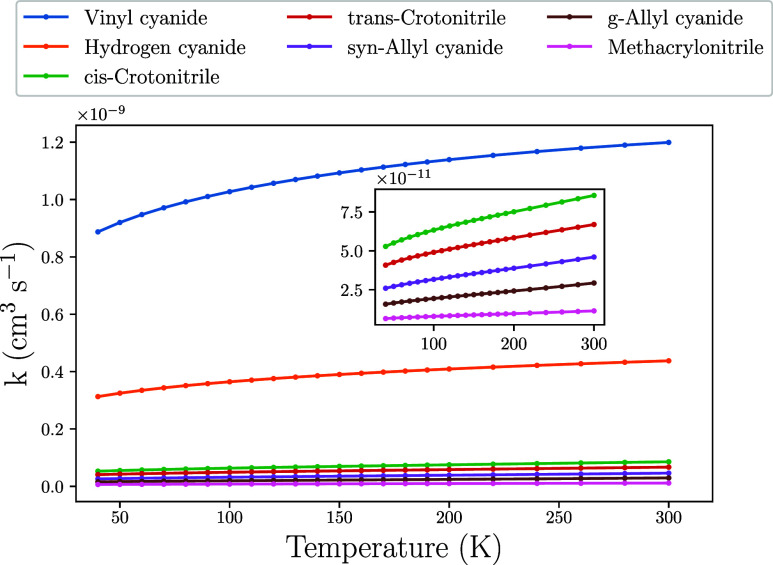
Evolution of the rate constants for the formation of **P1–P6** and **P13**.

**Table 3:** Rate Constants and Branching Ratios for the Formation of **P1–P6** and **P13** at *T* = 40 K and 10^–7^ atm

product	*k* (cm^3^ s^–1^)	branching ratio (%)
vinyl cyanide (P1)	8.9 × 10^–10^	66.1
*cis*-crotonitrile (P2)	5.3 × 10^–11^	3.9
*trans*-crotonitrile (P3)	4.0 × 10^–11^	3.0
methacrylonitrile (P4)	6.5 × 10^–12^	0.5
*syn*-allyl cyanide (P5)	2.6 × 10^–11^	1.9
*gauche*-allyl cyanide (P6)	1.6 × 10^–11^	1.2
hydrogen cyanide (P13)	3.1 × 10^–10^	23.2

Regarding the five isomers of cyanopropene that are produced with the elimination of hydrogen (**P2–P6**), we can find crotonitrile (CH_3_CHCHCN), allyl cyanide (CH_2_CHCH_2_CN) and methacrylonitrile (CH_3_C­(CN)­CH_2_). The attachment of the CN radical to the terminal – CH_2_ group to get crotonitrile (**P2** and **P3**) presents the higher rate constant. This is followed by the formation of allyl cyanide (**P5** and **P6**). The least favored process is the formation of methacrylonitrile (**P4**), involving capture in **R2** and H-elimination from the CH moiety, with a rate constant of 6.5 × 10^–12^ cm^3^ s^–1^ and branching ratio of around 0.5%. This is due to the competition with the formation of vinyl cyanide (**P1**), showing the higher efficiency of CH_3_ elimination over H-elimination. As we will show later in Section 4 we expect an underestimation of the branching ratio for the formation of **P4** as a consequence of this. The formation of hydrogen cyanide (**P13**) takes place with a rate constant of 3.1 × 10^–10^ cm^3^ s^–1^, and a branching ratio of 23.2%, becoming the second most favorable process after the formation of vinyl cyanide (**P1**). The formation of *gauche*-allyl cyanide (**P6**) through roaming (Figure 5) has a negligible rate constant, so that its formation only depends on the cyano-addition process.

The calculated branching ratios agree with the energetic profile shown in Figure 2a. The products **P2–P3** and **P5–P6** exhibit similar branching ratios because they are all directly accessible from the association complex **R1** through the submerged transition states **TS4-TS7**, which lie within a very narrow energy range (from −18.3 to −16.0 kcal mol^–1^). This energetic proximity explains the comparable ratios for their formation. Meanwhile, and as mentioned above, if the system enters via **R2** it has two possible pathways: the formation of vinyl cyanide (**P1**), which has the lowest exit barrier (−22.3 kcal mol^–1^) and the formation of methacrylonitrile (**P4**) with a higher competing barrier (−15.7 kcal mol^–1^, 6.6 kcal mol^–1^ above the one of the competing channel), which explains the low branching ratio for **P4**. Additionaly, we find the interwell isomerization (**R1** ↔ **R2**), which again is energetically more favored than the tautomerization mechanisms (**R1** ↔ **R9-R11**).

We fit the calculated rate constants into the Arrhenius equation (eq 19) for vinyl cyanide (**P1**), the isomers of cyanopropene (**P2–P6**) and hydrogen cyanide (**P13**) within a temperature range from 40 to 300 K. The fitted parameters are shown in Table 4 and they can be used to extrapolate the rate coefficients beyond the range of temperatures considered as shown in Figure S2 (Supporting Info).

**Table 4:** Arrhenius-Kooij Parameters of eq 8 for the Reactions Considered in Our Quantum Chemical Calculations^a^

product	α (cm^–3^ s^–1^)	β
vinyl cyanide (P1)	1.2 × 10^–9^	0.15
*cis*-crotonitrile (P2)	8.3 × 10^–11^	0.24
*trans*-crotonitrile (P3)	6.5 × 10^–11^	0.24
methacrylonitrile (P4)	1.1 × 10^–11^	0.29
*syn*-allyl cyanide (P5)	4.4 × 10^–11^	0.29
*gauche*-allyl cyanide (P6)	2.8 × 10^–11^	0.32
hydrogen cyanide (P13)	4.4 × 10^–10^	0.17

a The fit of eq 8 is done leaving γ = 0.

## 4 Discussion

We now analyze the branching ratios for the five detected isomers of cyanopropene (**P2–P6**), formed via CN addition followed by H elimination, excluding the formation of vinyl cyanide (**P1**) and hydrogen cyanide (**P13**), in order to compare them with observational results. Figure 8 shows the evolution of the calculated branching ratios for the formation of these cyanoderivatives with temperature. They have been extrapolated to lower temperatures (down to 10 K) to have a more rigorous comparison with the observational values in Table 5. The ratios that are derived from our rate constants agree reasonably with the observed ones (see Table 5), although we find some qualitative discrepancies. In both cases, *cis*-crotonitrile is found to be the most abundant isomer of cyanopropene. However, the observed column densities predict that it exists with an abundance three times higher than the *trans* isomer, while our calculations yield a ratio of 1.3:1. The *syn* and *gauche* isomers of allyl cyanide are also found to be in a similar ratio, however our calculations predict that the *syn* isomer is slightly more abundant.

**Table 5:** Comparison of the Calculated and Observed Abundance Ratios for Five Cyano Derivatives of Propene (**P2**-**P6**), Detected in Cernicharo et al.^6^,^a^

product	calculated ratio (%)	observed ratio (%)
*cis*-crotonitrile	37.4	30.2 ± 3.4
*trans*-crotonitrile	28.9	11.6 ± 1.2
methacrylonitrile	4.6	23.2 ± 2.2
*syn*-allyl cyanide	18.2	16.3 ± 1.6
*gauche*-allyl cyanide	10.9	18.6 ± 1.8

a The uncertainties in the observed ratios are derived from the propagation of the errors in the column densities reported in that work

**Figure 8:**
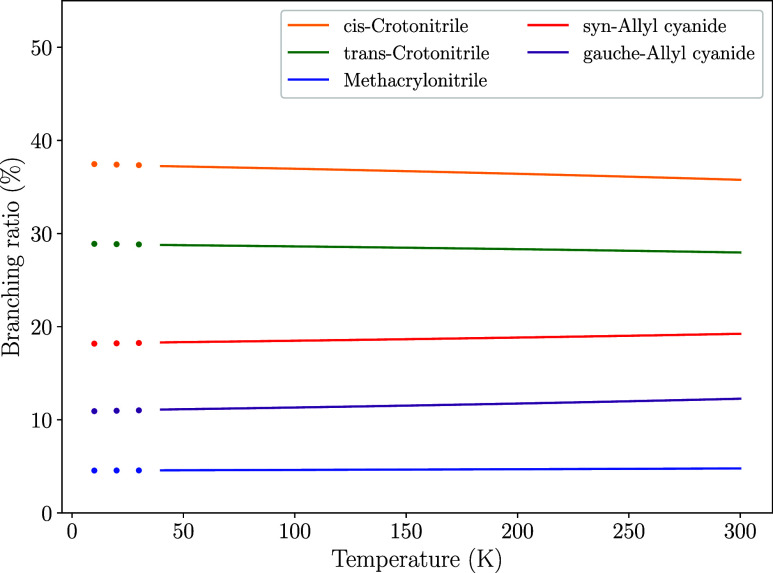
Branching ratios for the formation of cyanopropene isomers.

The most significant discrepancy arises from methacrylonitrile, which is predicted to have a lower branching ratio than the observations by a factor of approximately 5. It is interesting to hypothetisize the origin of the discrepancies with the observational constraints. The reason is multicausal. In the first place, we must consider the differential reactivity of the C_3_H_5_CN isomers after formation. As it was recently shown in García de la Concepción et al.^35^ different isomers can experience different ion–molecule reactivity in the gas phase, which can modulate the observed abundances. This reactivity is dependent to the relative stability of the charged species of the isomers, after a protonation or ionization, followed by reaction with a base, e.g., NH_3_ or e^–^. This mechanism is referred as the *sequential acid–base mechanism* ^35^ (SAB), and its study for cyanopropene is in our plans to fully explain the observational constraints of the different isomers. Another mechanism that could account for the discrepancies is the dissociative electron recombination (DR) of the protonated isomers. In this scenario, once the neutral isomers are formed, protonation followed by electron attachment could form high-energy isomers, especially considering the large exothermicities typically associated with DR reactions. However, it should be noted that the vast majority of DR branching ratios remain undetermined.^36^ While the SAB, or similar mechanisms like the relative dipole principle (RDP)^37^ are likely contributing factors to the inconsistencies observed among stereoisomers, they are unlikely to explain the specific case of methacrylonitrile (**P4**). This disagreement is more qualitative, making us suspect that it might arise from a limitation of our theoretical model, from additional formation channels, or can be an evidence for the role of DR for this species.

Additionally, we bring up two more hypotheses to explain the underestimation of the calculated branching ratio for methacrylonitrile. First, a monodimensional model for the capture of CN could be overestimating the formation of the competing **R1** with respect to **R2**. In the Supporting Information, we show that the isomerization between **R1** and **R2** is fast, thereby reducing the significance of differential capture. Second, as it is evinced in Figure 2, there are two elimination channels from **R2**, through the elimination of CH_3_ and H. Although the elimination of CH_3_ is energetically favorable, the elimination of H has a more pronounced effect of quantum tunneling, which in our model is treated only approximately by means of an Eckart barrier and within an RRKM framework. This approximation could be leading to an overestimation of the **P1** formation, and therefore a lower branching ratio for **P4**.

Among the isomeric forms of crotonitrile (CH_3_CHCHCN) and allyl cyanide (CH_2_CHCH_2_CN), Figure 9 shows how the branching ratios evolve with temperature for the *cis*-*trans* and *syn*-*gauche* isomers. Starting with the latter pair, we find that our simulations reproduce the observed branching ratio for the *syn* isomer within the observational uncertainties, while slightly underestimating the *gauche* isomer. The difference is below a factor 1.5 and therefore, it is unclear whether such a discrepancy can be attributed to uncertainty of our electronic structure solver. On the other hand, the ratio between *cis* and *trans*-crotononitrile is overestimated by a factor ∼ 2.0. In the case of this pair, the higher dipole moment of t-CH_3_CHCHCN (Dipole moments for all the products are collated in Table 6) is coherent with the RDP or SAB, and the observed ratio is likely to be affected by the ion–molecule reactivity of the C_3_H_5_CN isomers, as discussed above. At 40 K, our simulations show that *cis*-crotonitrile is formed with a branching ratio of 53.4% versus a 46.3% of *trans*-crotonitrile. In the case of allyl cyanide, the *syn* isomer is formed with a branching ratio of 62.3% versus a 37.7% for the *gauche* isomer. Although the branching ratios remain roughly constant with temperature, we can see that the *cis*/*trans* and *gauche*/*syn* ratios become slightly more balanced as the temperature rises. However, the differences are not significant.

**Figure 9:**
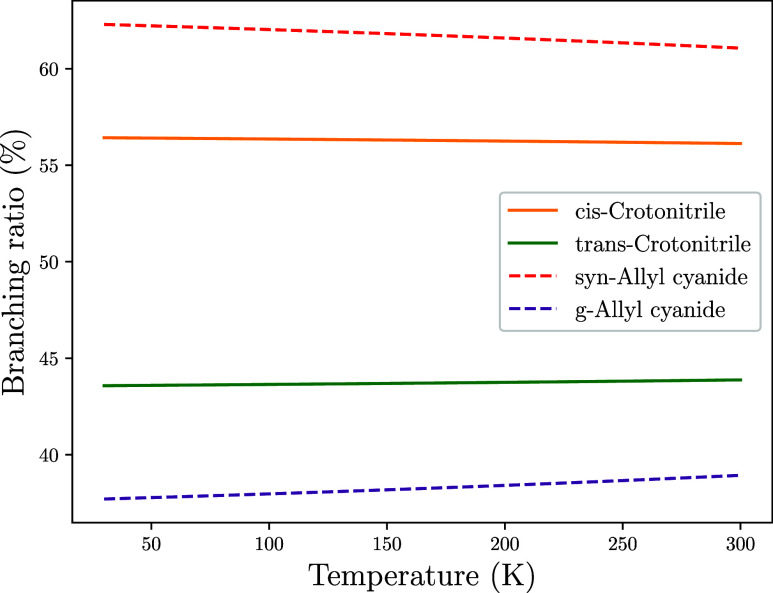
Branching ratios for the formation of one or another isomer of crotonitrile (solid lines) and allyl cyanide (dashed lines) from *T* = 40K to 300 K.

**Table 6:** Dipole Moments (μ) of the Five Cyano Derivatives (**P2**-**P6**) given in Debye, with Their Respective Components Along Axis a, b and c^a^

product	μ_a_	μ_b_	μ_c_	μ_total_
vinyl cyanide	4.21	0.73	0.0	4.27
*cis*-crotonitrile	4.16	1.82	0.0	4.54
*trans*-crotonitrile	5.03	0.57	0.0	5.06
*syn*-allyl cyanide	3.18	2.52	0.0	4.06
*gauche*-allyl cyanide	3.71	1.88	0.44	4.20
methacrylonitrile	4.27	0.33	0.0	4.28

a The values have been calculated at the revDSD-PBEP86­(D3BJ)/jun-cc-pVTZ level. For each column we provide the absolute value of each magnitude.

The calculated dipole moments are shown in Table 6, and they are generally in good agreement with the ones used for deriving the column densities in Cernicharo et al.^6^ The largest deviation was found for *trans*-crotonitrile (∼16%), which would affect the value of the column density by a ∼1.3 factor, but does not significantly affect the overall results.

Morales et al.^17^ determined the total rate constant for the title reaction, that from our theoretical results is calculated as the sum of all the individual rate constants calculated (*k* = ∑_*i*_ *k*_*i*_). The value of the total rate constant at 40 K is 1.3 × 10^–9^ cm^3^ s^–1^ that deviates from the experimental values by a factor of 3 at 40 K. An overestimation of the total rate constant is not surprising as our capture model is known to overestimate it, assuming exclusively that capture proceeds through the minimum energy path. Our results agree with Morales et al.^17^ in the main exothermic channels that are found to be accessible at low temperatures, although they differ in the branching ratios that were determined by Trevitt et al.^38^ and Trevitt et al.,^39^ summed to the contradictions with other experimental works.^40^ Therefore, our results support the qualitative view of Morales et al.^17^

Similarly, we have systematically compared our findings with previous theoretical studies. Huang et al.^15^ investigated the C_3_H_6_ + CN reaction channels using B3LYP/cc-pVTZ calculations with energy refinement at the CCSD­(T)/cc-pVTZ level. As summarized in Table 3, our results confirm that vinyl cyanide is the dominant product, with a branching ratio of 66.1%, in good agreement with the range of 70–86% reported by Huang et al.^15^ at zero collision energy. The main divergence between both studies concerns the minor reaction channels. Huang and co-workers concluded that only four of the five detected cyanoderivatives, namely crotonitrile, allyl cyanide, and products **P2**, **P3**, **P5**, and **P6**, turned out to be competitive. Although they described the formation of methacrylonitrile (**P4**) through an exothermic and accessible channel, they did not find it to be kinetically competitive. In contrast, our calculations indicate that methacrylonitrile can indeed be produced in small amounts through the investigated reaction. As discussed above, its branching ratio is likely underestimated in our analysis, but we cannot exclude the contribution of additional pathways to its formation. Regarding the H-abstraction channels, Huang et al.^15^ proposed that hydrogen cyanide could be formed without energetic constraints, but the absence of HCN in the experiments of Trevitt et al.^38^ led them to discard this possibility. Our results instead suggest that this channel may be viable, although the PES energetics are close to the limits of our theoretical accuracy. We therefore recommend that new experimental investigations be carried out to clarify the relevance of this pathway.

The comparison between the theoretical calculations and the experimental results is shown below in Table 7.

**Table 7:** Comparison of the Total Rate Constants and Branching Ratios for the Formation of Vinyl Cyanide (P1) from Different Studies^a^

work	*k* (cm^3^ s^–1^)	**P1** ratio
theoretical studies		
this work	1.3 × 10^–9^	0.66
Huang et al.^15^	-	0.70–0.86
experimental studies		
Morales et al.^17^	(3.9 ± 0.5) × 10^–10^	-
Gannon et al.^40^	1.73 × 10^–10^	-
Sims et al.^16^	(3.2 ± 0.3) × 10^–10^	-
Trevitt et al.^38^	-	0.75
Trevitt et al.^39^	-	0.59

a Only total rate constants (*k*) and branching ratios for the formation of the main product (vinyl cyanide, P1) are presented for clarity. The various studies report different types of data, such as rate constants measured at different temperatures or collision energies, and branching ratios for multiple products with differing degrees of isomer differentiation. A hyphen (-) indicates that the corresponding data are not reported in the original publication. The study of Gu et al.^18^ is not included because the crossed-beam experiments were performed with deuterated species, complicating direct comparison.

From an astrochemical perspective, our calculations suggest that the formation of cyanopropene can be explained from the C_3_H_6_ + CN reaction, as proposed in Cernicharo et al.^6^ This is further confirmed by the calculation of cyanopropene abundances in our gas-phase astrochemical models (Supporting Information) where the inclusion of our theoretically derived rate constants evidence a good match between models and observations. Besides, the sensitivity of the prediction of C_3_H_5_CN to the C/O ratio is an additional evidence of the importance of the gas-phase chemistry for this reaction. This carries important implications for the chemistry of cyanopropene in particular and of nitrogen-bearing complex organic molecules (N-COMs) in cold interstellar clouds. First, it supports a pure gas-phase mechanism in the formation of cyanopropene, following the example of other recently detected cyanides.^2,4^ The chemical complexity of CN bearing molecules can be traced back to the reactivity of CN radicals with unsaturated carbons as discussed in the Introduction. However, in recent theoretical work^41–43^ the reactivity of CN on ices favors hydrogenation and reaction with the ice, hindering the formation of N-COMs different than methylamine (CH_3_NH_2_).

Moving up the chemical complexity ladder makes it increasingly difficult to extract theoretical rate constants for the formation of larger N-COMs. This challenge arises both from the high computational cost required for an accurate description of the PES energetics and from the large number of stationary points that must be considered in the kinetic analysis. In this work, using an accurate approach, we have shown how branching ratios are strongly influenced by subtle energy differences in the PES, as discussed above. The most important methodological conclusion, however, is that while the formation of the detected C_3_H_5_CN isomers can be exclusively attributed to the C_3_H_6_ + CN reaction, the quantitative reproduction of the observed ratios requires additional input in the form of ion–molecule destruction channels that may interconvert the isomers (see, e.g., García de la Concepción et al.^35^ or Shingledecker et al.).^37^ We aim to continue addressing these gaps in the isomeric ratios of such molecules in future work.

## 5 Conclusions

We present an accurate theoretical investigation of the gas-phase reaction between the cyano radical (CN) and propene (C_3_H_6_) under interstellar conditions. Given the low temperatures and pressures of the ISM, only exothermic reactions with submerged energy barriers are feasible. The reaction proceeds through the formation of two initial association complexes, CH_3_CHCH_2_CN and CH_3_CH­(CN)­CH, which can interconvert via a triangular intermediate or undergo tautomerization. The dominant pathways lead to the production of vinyl cyanide (**P1**), five cyano derivatives (**P2–P6**), and hydrogen cyanide (**P13**) through H atom elimination. In contrast, the formation of isocyano derivatives is energetically disfavored, with barriers too high to be overcome at low temperatures, and can thus be ruled out under ISM conditions. Rate constants derived using an AITSME approach yield branching ratios that can be directly compared with astronomical observations.^6^ While the results reproduce the dominant formation of vinyl cyanide, some discrepancies remain in the relative ratios of the minor products. These findings suggest that additional destruction mechanisms, particularly ion–molecule processes capable of interconverting isomers, may play a key role in shaping the observed abundances. This highlights the importance of incorporating ion–molecule destruction chemistry into the quantitative astrochemistry of isomerism.

## Supplementary Material



## Footnotes

† Q-band Ultrasensitive Inspection Journey to the Obscure TMC-1 Environment.

‡ Information on how to run calculations using this functional with different electronic structure codes can be found at https://www.compchem.me/revdsd-pbep86-functional

## Notes

Summary of the stationary points of the C_3_H_6_ + CN reaction, link to the Zenodo repository where the Cartesian coordinates of each stationary point are stored. Discussion on capture vs isomerization in the kinetics of the reaction. Astrochemical Modeling (PDF)
