# Supplementary material for: Isomer-Specific Branching Ratios in the Formation of Cyanopropene (C3H5CN) through the C3H6 + CN Reaction under Interstellar Conditions
Source: ACS Earth Space Chem. 2025 Nov 6;9(11):2747–58. doi: 10.1021/acsearthspacechem.5c00274 (PMC12643045; doi:10.1021/acsearthspacechem.5c00274)
Supplement: Supplementary file 1 [file sp5c00274_si_001.pdf]

Supporting Information: Isomer-Specific Branching Ratios in the Formation of Cyanopropene (C_3H_5CN) through the C_3H_6 + CN Reaction under Interstellar Conditions

María Mallo,* Marcelino Agúndez, Carlos Cabezas, José Cernicharo, and Germán Molpeceres

[1] *Instituto de Física Fundamental, CSIC, C/ Serrano 123, 28006 Madrid, Spain*

E-mail: german.molpeceres@iff.csic.es

Contents

1 Summary of the stationary points of the $C_3H_6 + CN$ reaction. S-2

2 Competition between capture and isomerization in the kinetics of the reaction S-4

3 Astrochemical Modelling S-5

References S-6

1 Summary of the stationary points of the C_3H_6 + CN reaction.

Table S1: Relative energies of the stationary points of the C_3H_6 + CN reaction. The energies are given in kcal mol^{-1} and the zero-point vibrational energy (ZPVE) corrections are included.

Label	ΔU	Label	ΔU	Connection
Reactants	0.0	TS1	-30.4	R1-R7
R1	-54.6	TS2	-30.3	R7-R2
R2	-52.0	TS3	-22.3	R2-P1
R3	-32.0	TS4	-18.3	R1-P2
R4	-31.1	TS5	-18.1	R1-P3
R5	-17.0	TS6	-16.9	R1-P5
R6	-19.2	TS7	-16.0	R1-P6
R7	-31.8	TS8	-15.7	R2-P4
R8	9.8	TS9	-25.5	R9-P1
R9	-61.1	TS10	-19.6	R9-P2
R10	-60.8	TS11	-19.5	R9-R1
R11	-52.7	TS12	-19.1	R10-R1
R12	-38.4	TS13	-19.1	R10-P3
R13	-30.3	TS14	-15.2	R11-P5
R14	-41.9	TS15	-14.6	R11-P6
R15	-40.4	TS16	-13.8	R11-R1
P1	-31.6	TS17	-2.8	R2-R5
P2	-23.8	TS18	-4.9	R1-R6
P3	-23.5	TS19	-4.5	R4-R6
P4	-23.0	TS20	-4.0	R3-R5
P5	-19.8	TS21	-0.8	R1-R3
P6	-19.0	TS22	0.1	R4-P7
P7	-9.8	TS23	0.95	Reac-R4
P8	-2.4	TS24	1.64	Reac-R3
P9	-1.4	TS25	2.5	R2-R4
P10	-0.8	TS26	4.3	R3-P9
P11	2.8	TS27	4.6	R3-P10

Continued on next page

Table S1 – continued from previous page

Label	ΔU	Label	ΔU	Connection
P12	3.7	TS28	5.2	R4-P8
P13	-39.7	TS29	5.8	R3-P11
		TS30	6.8	R3-P12
		TS31	10.1	R4-R8
		TS32	10.8	R3-R8
		TS33	-3.4	R12-P7
		TS34	2.4	R12-P9
		TS35	6.1	R3-R12
		TS36	7.5	R13-P11
		TS37	8.2	R13-P12
		TS38	8.4	R3-R13
		TS39	-22.7	R14-R15
		TS40	-11.8	R15-P6

All the XYZ coordinates of the stationary points shown in this work can be retrieved in the Zenodo repository:

`https://zenodo.org/records/17434343`

2 Competition between capture and isomerization in the kinetics of the reaction

As mentioned in the main text, phase-space or classical capture theory overestimate the total rate constant. To contextualize this overestimation, we analyzed the kinetics of the different entrance channels, as the initial capture leading to **R1**, **R2** or **R14** is decisive for the final product distribution. Among these, capture into **R1** is the most favorable, with a branching ratio of 39.8%, closely followed by **R2** at 36.9%. Capture into **R14**, which corresponds to the abstraction channel, accounts for 23.3%, representing a non-negligible contribution. These results indicate that the capture into **R1** and **R2** are comparably favorable and clearly dominant over the abstraction pathway, in agreement with the ratios reported in the text.

In order to evaluate the influence of the entrance channels on the final product branching ratios, we also examined the **R1** ↔ **R2** isomerization process, which exhibits a unimolecular rate constant on the order of 10^8 s^{-1}, against the direct evolution from **R1** and **R2** to products. This rate was compared with those of the product formation channels, as shown in Figures S1 and S2. The microcanonical isomerization rate at zero collision energy (the energy of the reactant asymptote) is, in all cases, higher than the product formation rates, indicating that the system can interconvert between the two intermediates rather than proceeding directly to products. Consequently, the initial capture into **R1** or **R2** is secondary in the final product distribution, as both intermediates contribute to the product formation. The most competitive product channel corresponds to the formation of vinyl cyanide (P1) in Figure S2, which exhibits a rate constant similar to that of the isomerization at higher collision energies, not relevant for the present, low-temperature study.

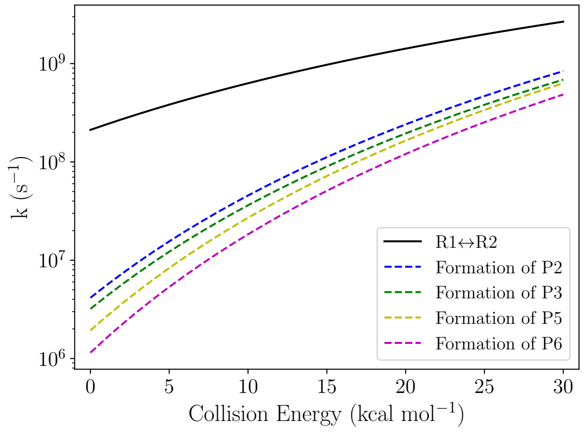

Figure S1: Unimolecular rate constants for the isomerization **R1** ↔ **R2** vs. the direct evolution to products from **R1** with respect to collision energies.

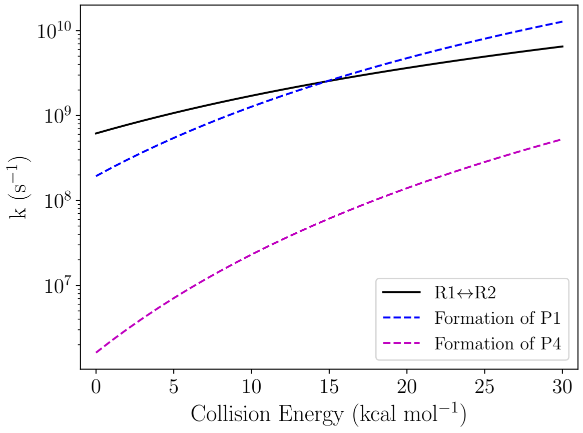

Figure S2: Unimolecular rate constants for the isomerization **R1** ↔ **R2** vs. the direct evolution to products from **R2** with respect to collision energies.

3 Astrochemical Modelling

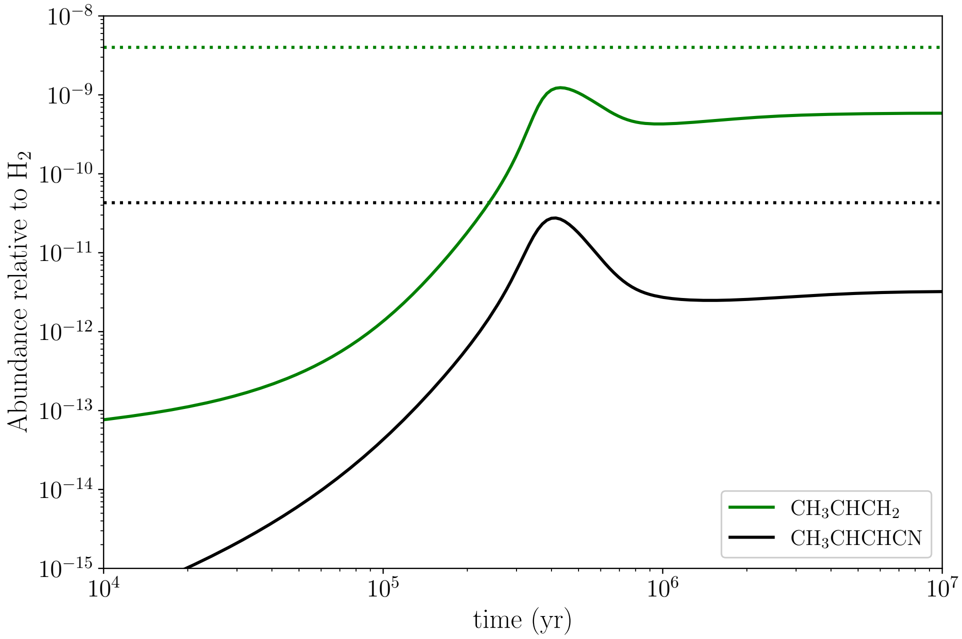

Figure S3: Abundance of cyanopropene (with no isomer differenciation) and propene. The solid lines represent the predicted abundances with our calculated rate constants and the dashed lines the observational values.

Astrochemical models are used to discern the overall contribution of the reaction C_3H_6 + CN for the formation of the cyanoderivatives **P2-P6**, which are detected in TMC-1 with a well-known abundance. We construct a time-dependent gas-phase chemical model with the typical parameters of cold dense clouds, consisting of a volume density of H nuclei of 2×10^4 cm^{-3}, a temperature of 10 K, a cosmic-ray ionisation rate of H_2 of 1.3×10^{-17} s^{-1} and a visual extinction of 30 mag.[S1] The gas-phase chemical network is based on the RATE22 network from the UMIST database[S2] and we adopt the so-called "low-metal" elemental abundances.[S1] The abundance of oxygen is varied to ensure a C/O ratio of 1, which results in a better overall agreement between calculated and observed abundances in TMC-1.[S3]

Current models significantly underestimate the abundance of propene in TMC-1,[S4] denoting that the proposed gas-phase reactions alone are insufficient to explain its observed abundance. Then, to evaluate the impact of the calculated rate constants, we have truncated the synthesis pathway to propene in our model by introducing a sequence of reactions designed to enhance the abundance of propene. Specifically, we considered the sequence $C_3H_3^+ + H_2 \rightarrow C_3H_5^+ + H_2 \rightarrow C_3H_7^+ + e^- \rightarrow C_3H_6$ with rate coefficients that are selected to artificially reproduce the observed abundance of propene.[S5] Nevertheless, it should be noted that these rate constants are not realistic and the problem of propene formation in cold dense clouds remains open. This approach is therefore only useful as a tool to evaluate the influence of our calculated

rate constants on astrochemical models with greater rigor.

Figure S3 shows the time evolution of the predicted abundances of propene and cyanopropene (with no isomer differentiation) in our model of TMC-1. We can see that an observed propene/cyanopropene ratio of ~ 100, which agrees with our calculations. This consistency indicates that the calculated rate constants explain the relative abundance of cyanopropene in TMC-1, supporting the conclusion that the title reaction is a major pathway for the formation of cyanopropene in this source.

References

(S1) Agúndez, M.; Wakelam, V. Chemistry of dark clouds: databases, networks, and models. *Chemical Reviews* **2013**, *113*, 8710–8737.

(S2) Millar, T.; Walsh, C.; Van de Sande, M.; Markwick, A. The UMIST database for astrochemistry 2022. *Astronomy & Astrophysics* **2024**, *682*, A109.

(S3) Agúndez, M.; Cernicharo, J. in preparation.

(S4) Lin, Z.; Talbi, D.; Roueff, E.; Herbst, E.; Wehres, N.; Cole, C. A.; Yang, Z.; Snow, T. P.; Bierbaum, V. M. Can interstellar propene (CH3CHCH2) be formed via gas-phase reactions? *The Astrophysical Journal* **2013**, *765*, 80.

(S5) Marcelino, N.; Cernicharo, J.; Agúndez, M.; Roueff, E.; Gerin, M.; Martín-Pintado, J.; Mauersberger, R.; Thum, C. Discovery of interstellar propylene (CH2CHCH3): Missing links in interstellar gas-phase chemistry. *The Astrophysical Journal* **2007**, *665*, L127.
